# Supplementary material for: Transcriptome Profiling of Vero E6 Cells during Original Parental or Cell-Attenuated Porcine Epidemic Diarrhea Virus Infection
Source: Viruses. 2023 Jun 23;15(7):1426. doi: 10.3390/v15071426 (PMC10386749; doi:10.3390/v15071426)
Supplement: Supplementary file 1 [file viruses-15-01426-s001.zip › Supplementary Materials.docx]

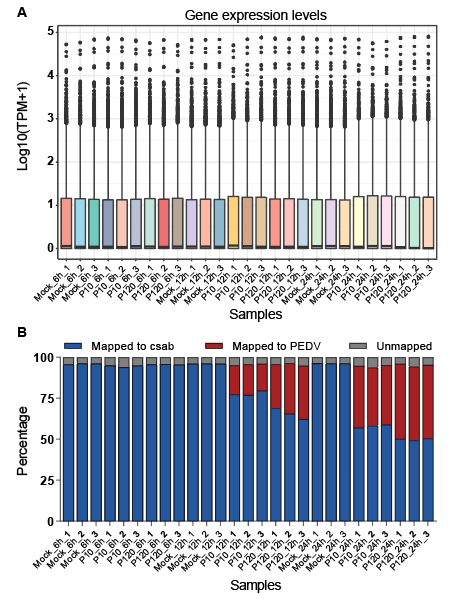


Supplementary figure S1

Basic information of sequencing reads. A. Box plot of gene expression levels. The x-axis is the name of the sample, and the y-axis is the value of the expression TPM after log 10 logarithmic processing. Each color in the figure represents a sample. B. Histogram of reads alignment distribution.


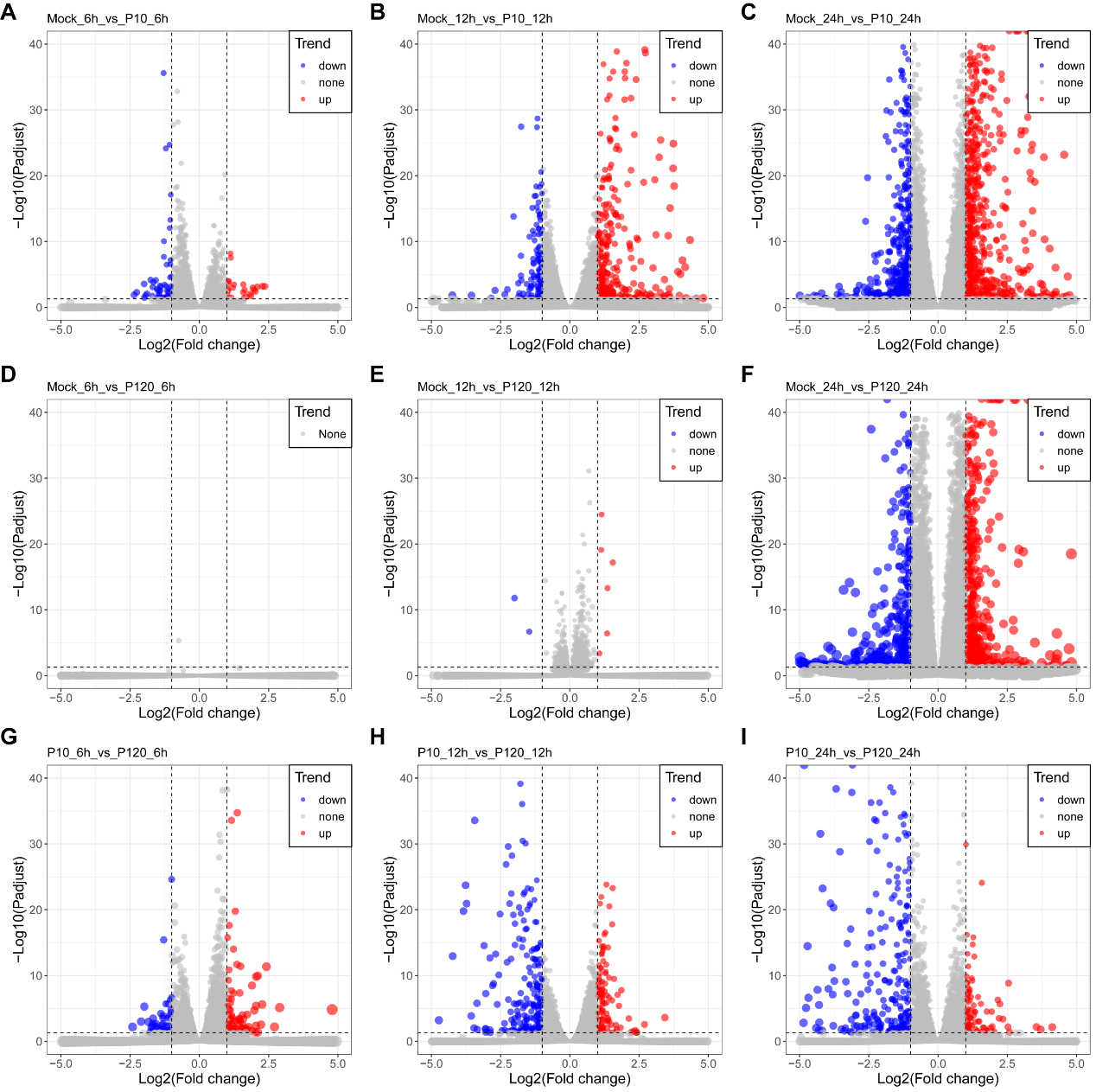


Supplementary figure S2

Volcano plots of DEGs. A-C. Volcano plots of DEGs between CT-P10 infected groups and mock-infected groups at 6, 12 and 24 hpi. D-F. Volcano plots of DEGs between CT-P120 infected groups and mock-infected groups at 6, 12 and 24 hpi. G-I. Volcano plots of DEGs between CT-P120 infected groups and CT-P10 infected groups at 6, 12 and 24 hpi.


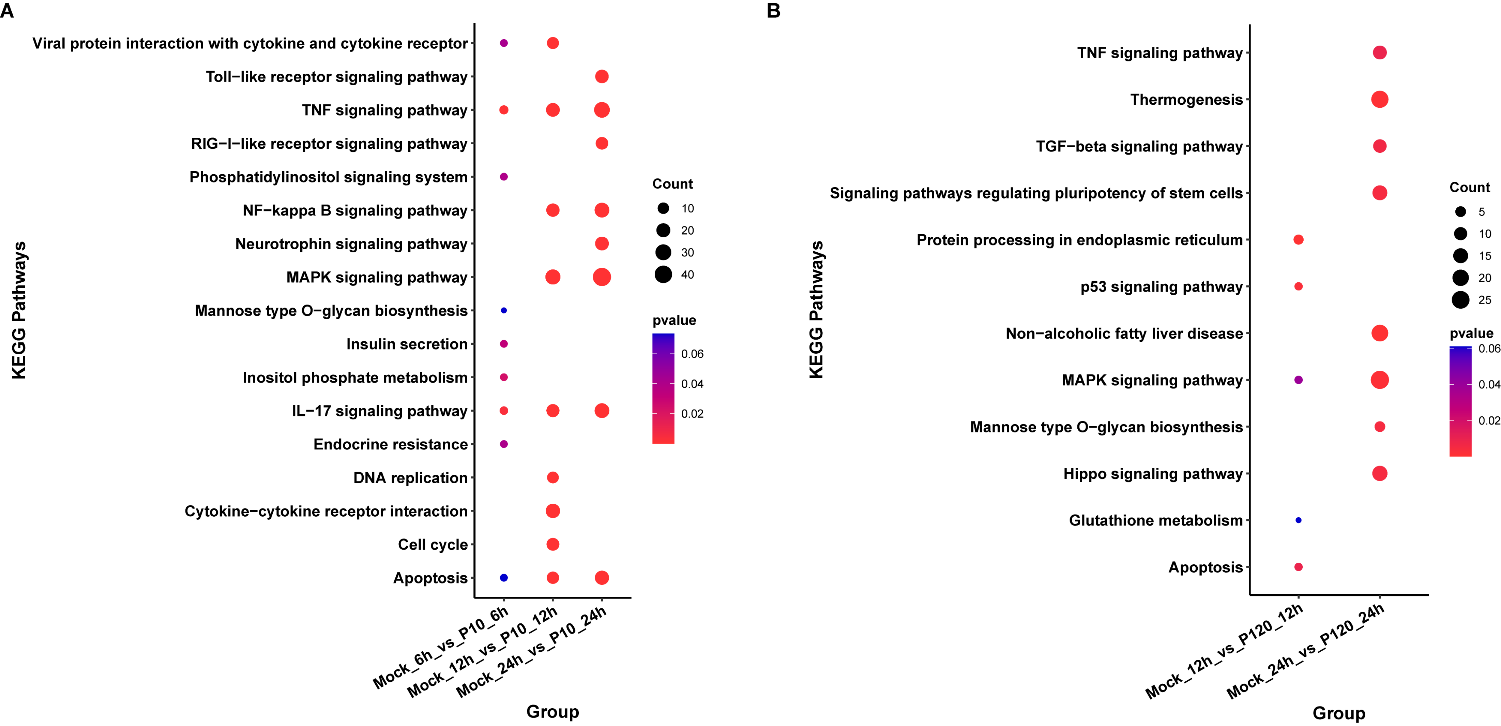


Supplementary figure S3

Bubble plots of KEGG pathways between CT-P10 or CT-P120 infected group and mock infected group. A. Bubble plot of KEGG pathways in CT-P10 infected groups compared with Mock-infected groups. B. Bubble plot of KEGG pathways in CT-P120 infected groups compared with Mock-infected groups. The size of each point represents the number of DEGs, the color of each point represents the p-value of enriched KEGG pathways.


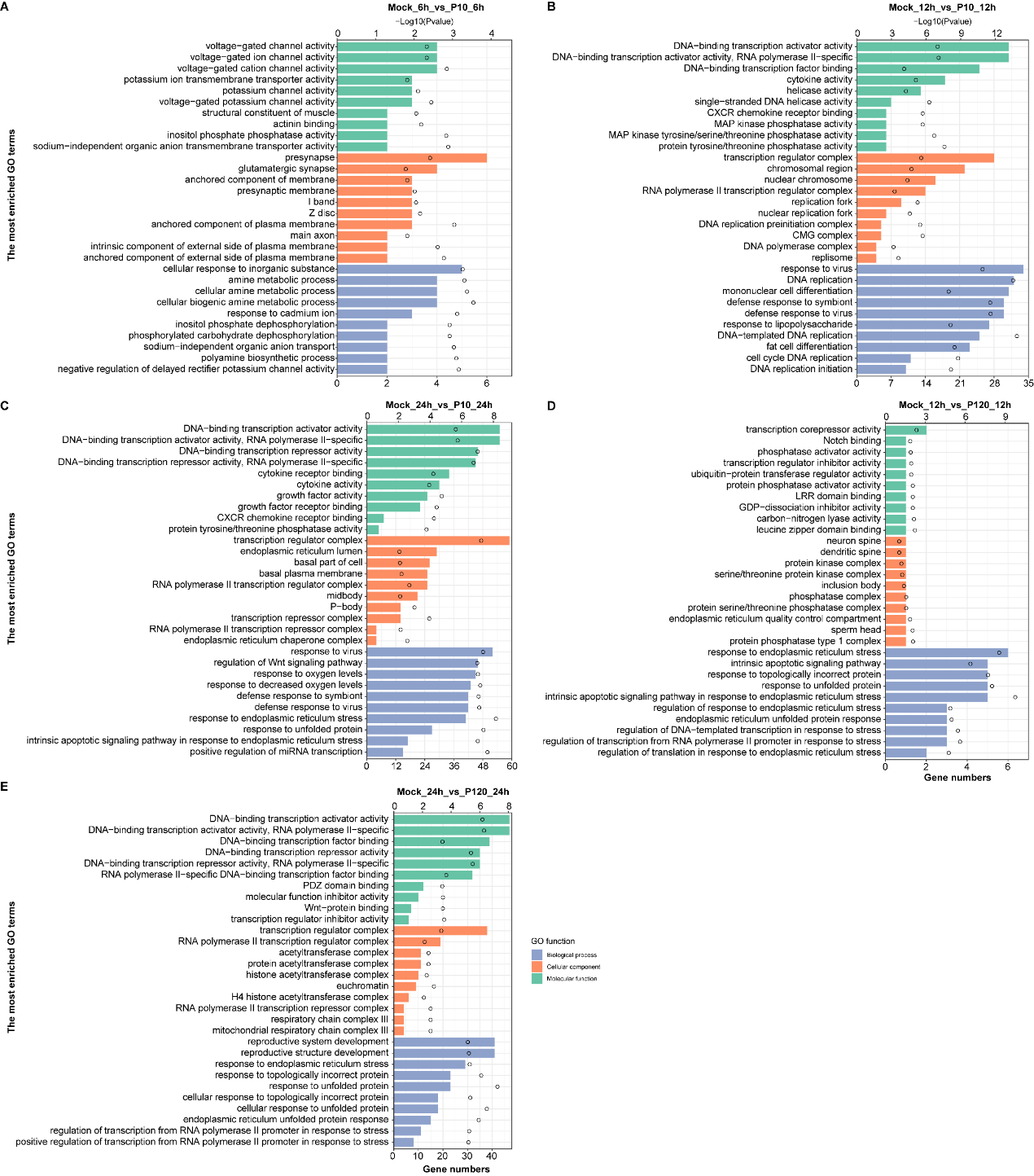


Supplementary figure S4

Bar plots of Gene Ontology (GO) terms between CT-P10 or CT-P120 infected group and mock infected group. A-C. Bar plot of GO terms in CT-P10 infected groups compared with Mock-infected groups at 6, 12 or 24 hpi. D-E. Bar plot of GO terms in CT-P120 infected groups compared with mock-infected groups at 12 or 24 hpi. Bars in green indicated biological process terms, bars in orange indicated cellular component terms, bars in blue indicated molecular function terms. X-axis in bottom indicated gene numbers in each GO terms, x-axis in top indicated p-value of GO terms, y-axis indicated different GO terms.


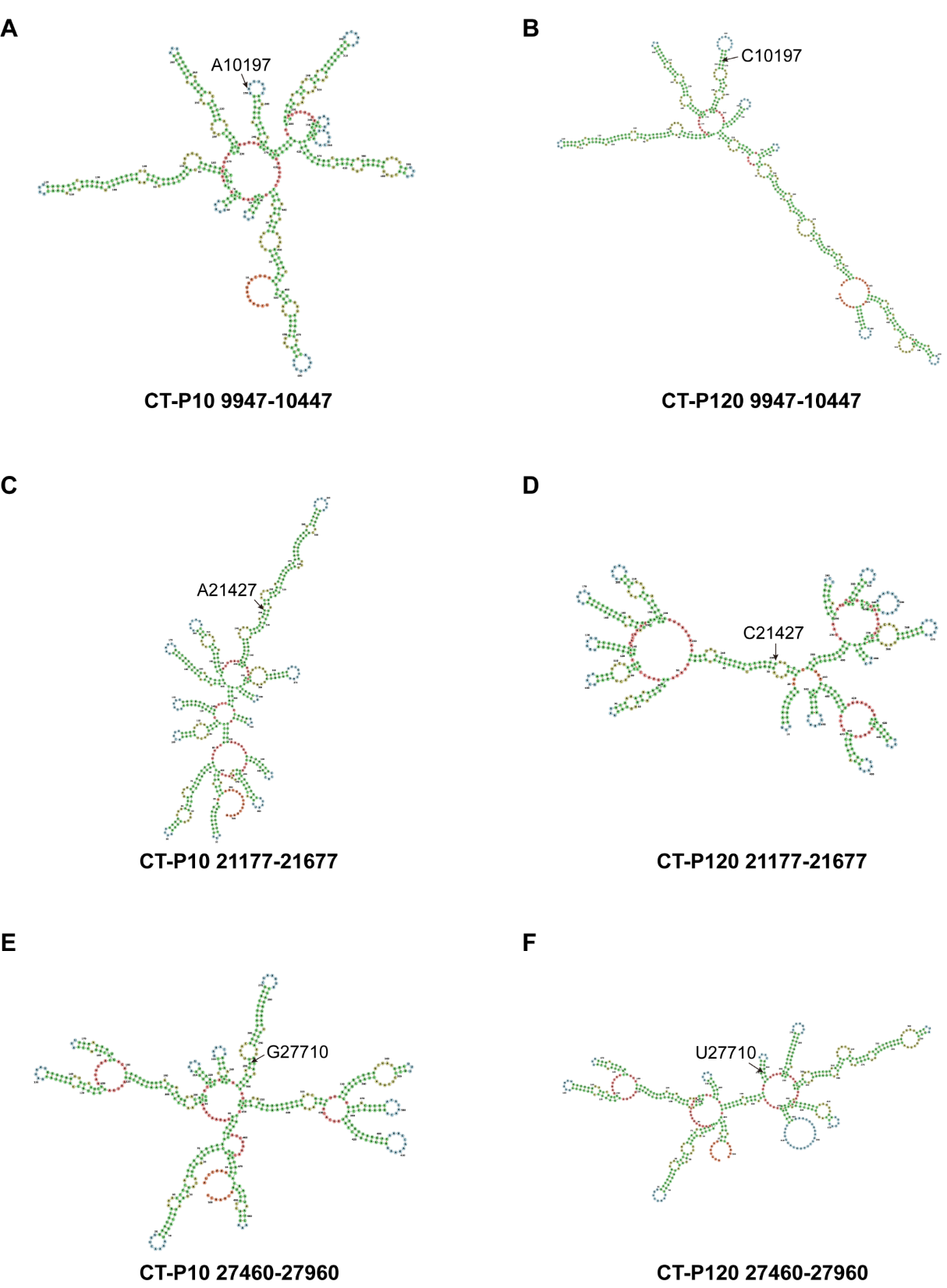


Supplementary figure S5

RNA secondary structure flanking the CT-P120 genome three mutation region (250 bp upstream and 250 bp downstream of mutation site). A, C and E. RNA secondary structure of CT-P10 genome flanking the mutation region at 10197, 21427 and 27710. B, D and F. RNA secondary structure of CT-P120 genome flanking the mutation region at 10197, 21427 and 27710.


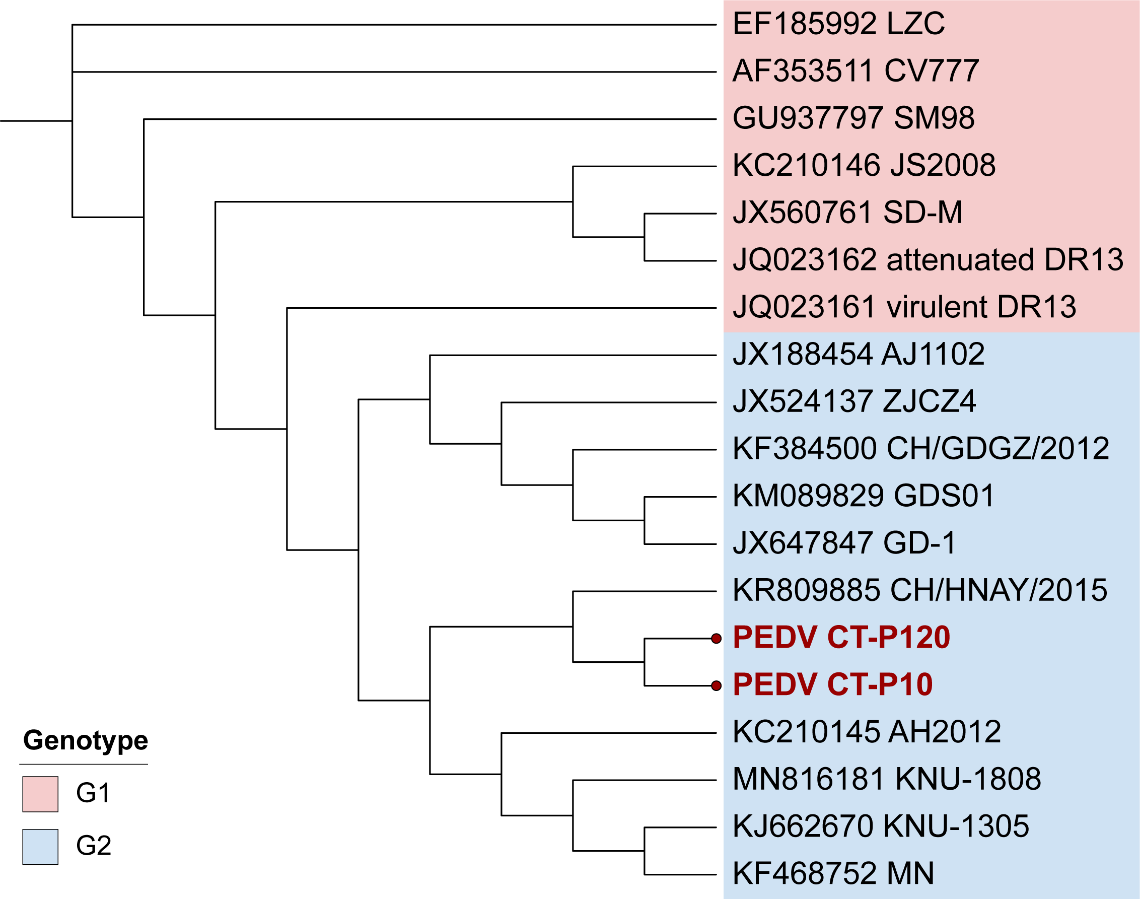


Supplementary figure S6

Phylogenetic analysis of CT-P10 and CT-P120 strains based on whole genome.
