# Supplementary material for: Transcriptome Profiling of Vero E6 Cells during Original Parental or Cell-Attenuated Porcine Epidemic Diarrhea Virus Infection
Source: Viruses. 2023 Jun 23;15(7):1426. doi: 10.3390/v15071426 (PMC10386749; doi:10.3390/v15071426)
Supplement: Supplementary file 1 [file viruses-15-01426-s001.zip › Tables and Figures.docx]

Table 1. Sequences of all primers used in this study.

| Gene | Primer | Sequence | Product length (bp) |
| --- | --- | --- | --- |
| TRAF3 | Forward | 5'-CCTTGTTCCGATTTGGAGGTG-3' | 300 |
|  | Reverse | 5'-TGACCCGGCTCCATTCTGTG-3' |  |
| IRF3 | Forward | 5'-TTGTGACCTCAGGAGTTGGG-3' | 249 |
|  | Reverse | 5'-GCTTCAGTGGGTTTTCACGG-3' |  |
| IFNL1 | Forward | 5'-CGGGAATTGGGACCTAAGGC-3' | 274 |
|  | Reverse | 5'-GCCAGGGGACTCCTTTTCGG-3' |  |
| ISG15 | Forward | 5'-CACGGCCATGGGTAGGGA-3' | 266 |
|  | Reverse | 5'-TCCTCACCAGGATGCTCAGT-3' |  |
| NFKB1 | Forward | 5'-GGCTACCCTGGCACAGAAAT-3' | 291 |
|  | Reverse | 5'-TCATCCCGGAGCTCGTCTAT-3' |  |
| MAP2K3 | Forward | 5'-CCATCGGAGACAGGAACTTTGA-3' | 260 |
|  | Reverse | 5'-GACGTCCAAGTCCATGAGCA-3' |  |
| IL1A | Forward | 5'-GCCCGCAATCAAAGCATCAT-3' | 217 |
|  | Reverse | 5'-GTGTCTCAGGCAGCTCCTTC-3' |  |
| CCL2 | Forward | 5'-GTGTCCTAAAGAAGCAGTGATCTTC-3' | 198 |
|  | Reverse | 5'-TCTGAGGGTATTTAGGGCAAGT-3' |  |
| GAPDH | Forward | 5'-CGGAGTGAACGGATTTGGC-3' | 248 |
|  | Reverse | 5'-CACCCCATTTGATGTTGGCG-3' |  |

Table 2. Numbers of genes clustered in six WGCNA modules.

| Module | blue | yellow | brown | turquoise | green | red |
| --- | --- | --- | --- | --- | --- | --- |
| Gene number | 1154 | 923 | 926 | 1470 | 238 | 48 |


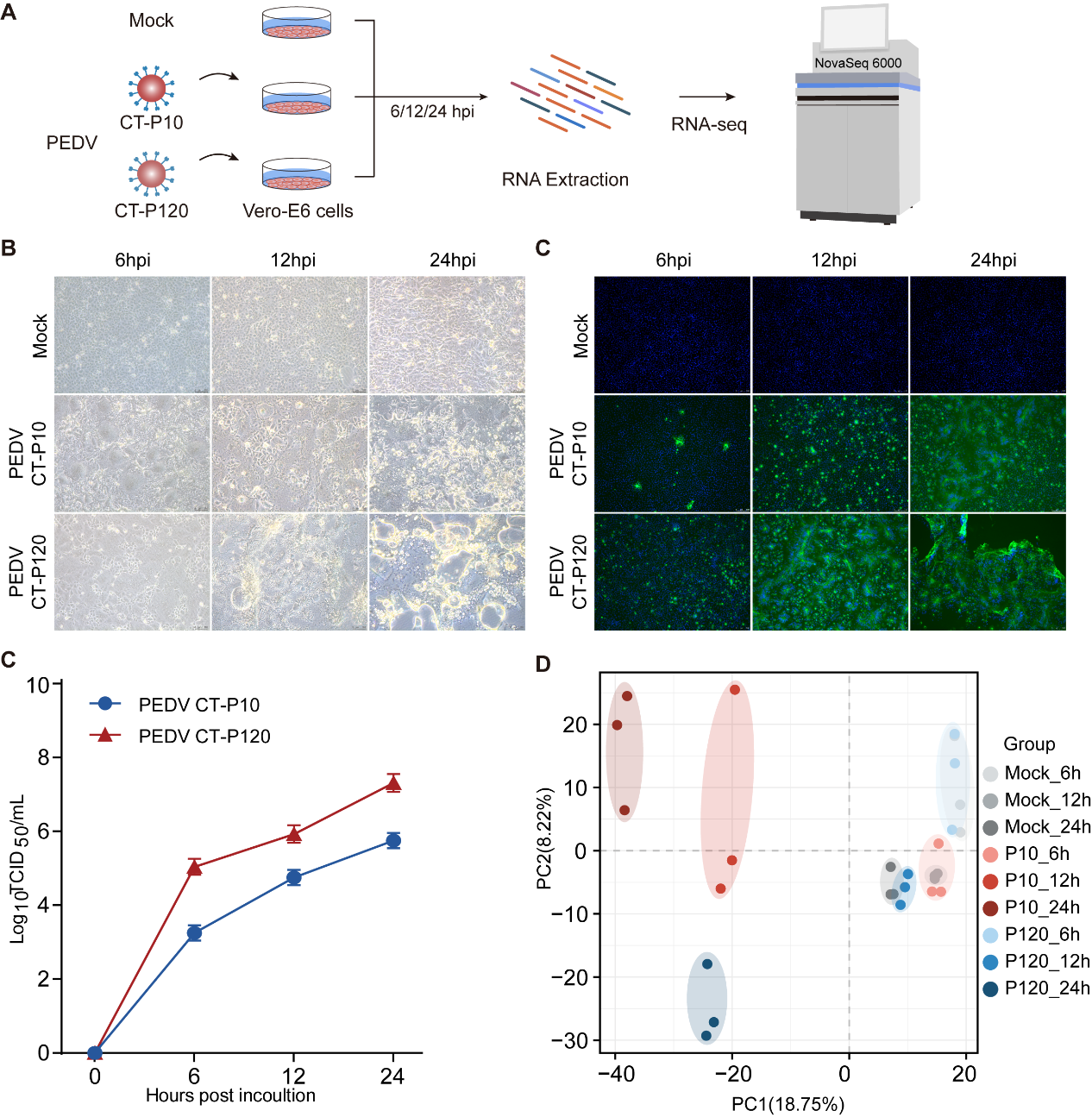


Figure 1

Basic information of this study. A. Diagram of RNA-seq experimental design. B. Cells were observed under a bright field through microscope. C. Identification of PEDV infection by indirect immunofluorescence. The nucleus was stained by 4',6-diamidino-2-phenylindole (DAPI), blue; the PEDV N protein was stained by Fluorescein isothiocyanate (FITC), green. D. PEDV proliferation curve determined using TCID_50_. E. Principal component analysis of each sample.


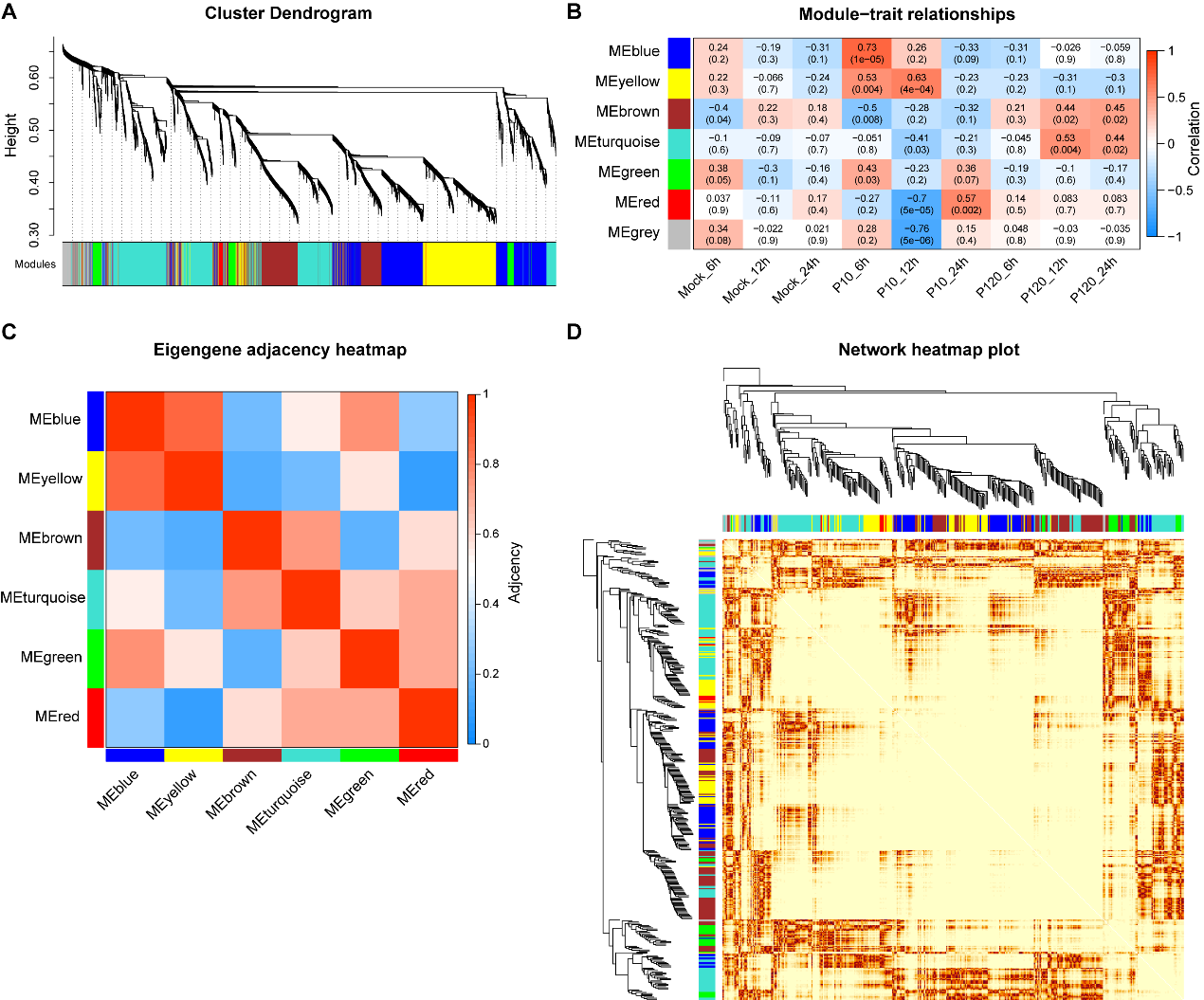


Figure 2

Weighted correlation network analysis (WGCNA). A. Genes are divided into modules according to the expression trend of genes, where branches represent a gene, a color represents a module, and if the color is gray, it represents a gene that is not assigned to a specific module. B. Show the correlation size between modules and modules, each column or row represents a module, the color in the figure represents the correlation size between modules, red represents the greater correlation between modules, and green represents the small correlation between modules. On the left or above is a tree view of module clustering, and the closer the two module branches are, the more related the two modules are. C. The x-axis represents different groupings, the y-axis represents different modules, the number on the left side of the figure indicates the number of genes in the module, each set of data on the right represents the correlation coefficient and significance of the module with the phenotype and the significance P value (in parentheses), red represents the positive correlation between the module and the phenotype, and blue represents the negative correlation between the module and the phenotype. C. Module eigengene adjacency heatmap. Module-eigengenes (ME) in this analysis are defined as the first principal component of a coexpression module matrix. The heatmap shows the relatedness of the 13 co-expression modules (ME1-ME13) identified by WGCNA with red being highly related and blue being not related.


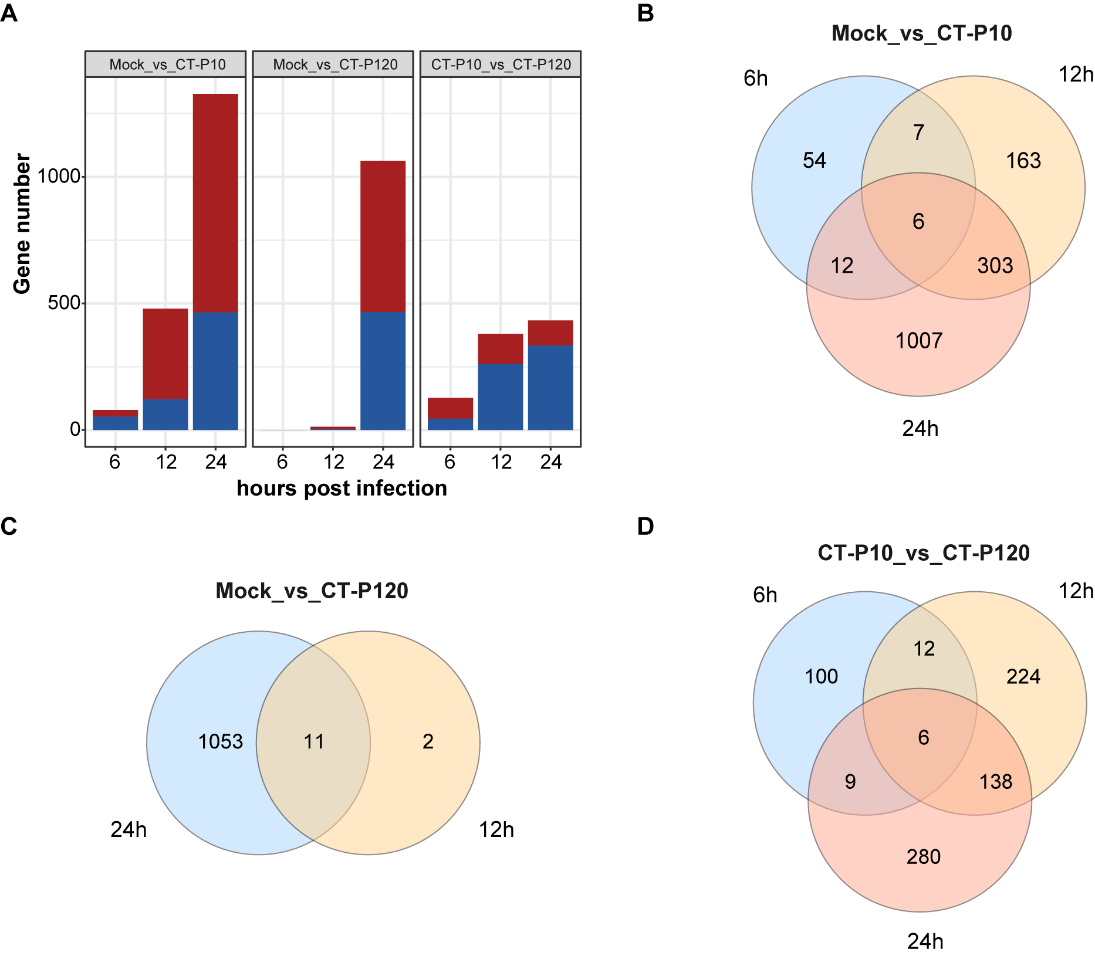


Figure 3

Analysis of differentially expressed genes. A. Histograms of differentially expressed genes number. Bars in blue indicated down-regulated genes numbers, bars in red indicated up-regulated genes number. B. Venn diagrams of differentially expressed genes between mock and CT-P10 infection groups at three time points. C. Venn diagrams of differentially expressed genes between mock and CT-P120 infection groups at two time points. D. Venn diagrams of differentially expressed genes between CT-P10 and CT-P120 infection groups at three time points.


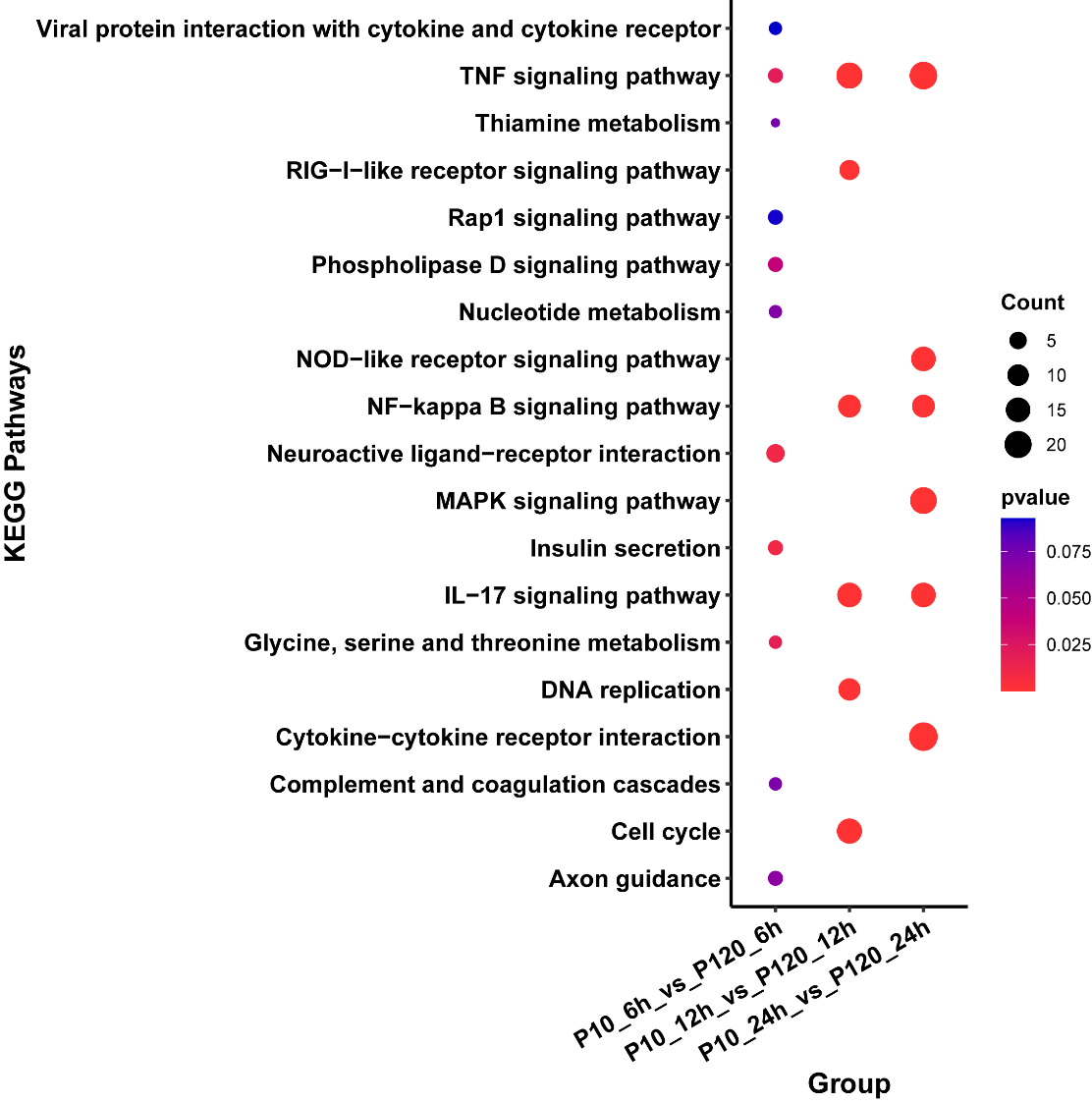


Figure 4

Bubble plot of KEGG pathways in CT-P120 infected groups compared with CT-P10 infected groups. The size of each point represents the number of DEGs, the color of each point represents the p-value of enriched KEGG pathways.


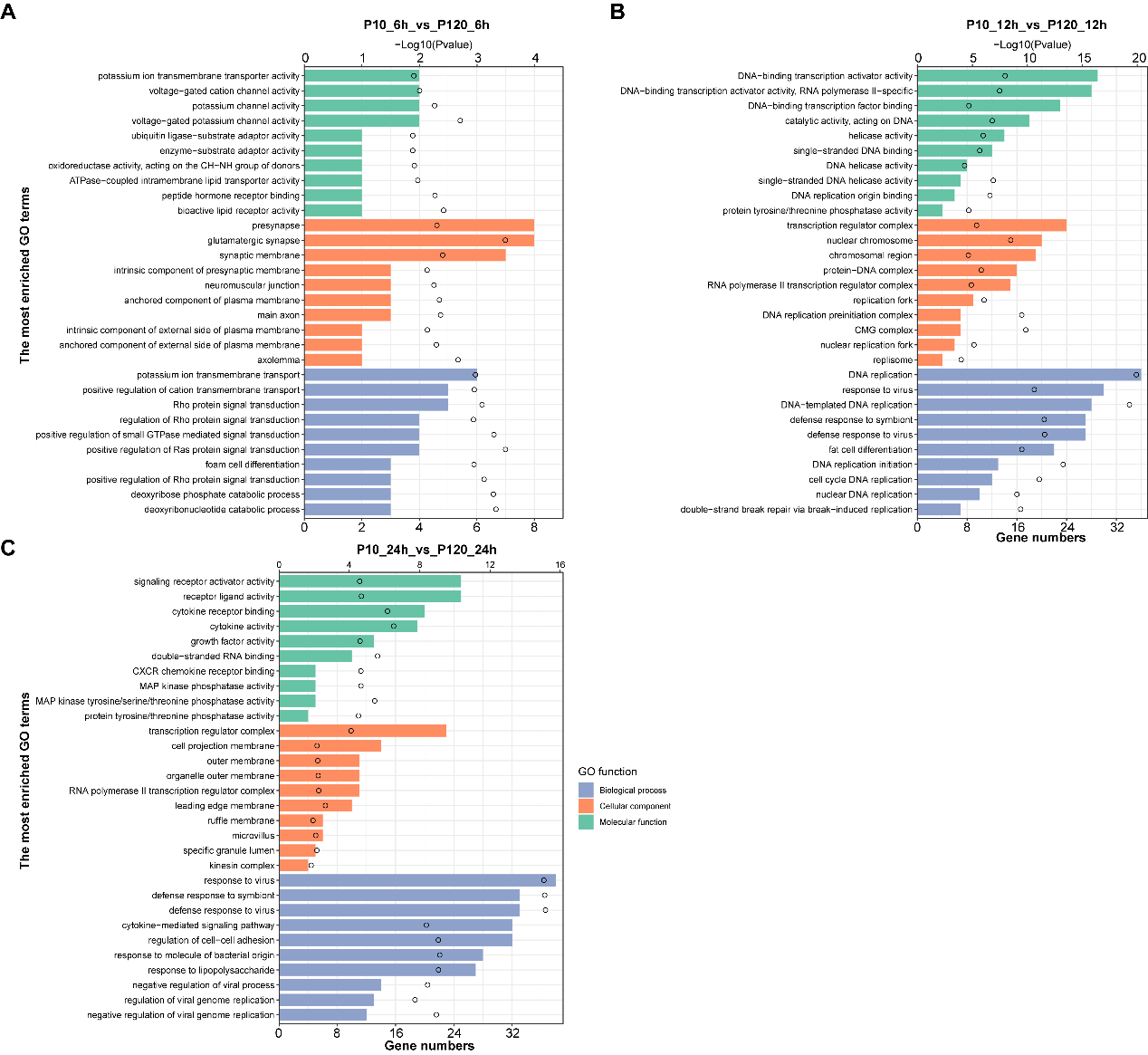


Figure 5

Bar plot of GO terms in CT-P120 infected groups compared with CT-P10 infected groups. Bars in green indicated biological process terms, bars in orange indicated cellular component terms, bars in blue indicated molecular function terms. X-axis in bottom indicated gene numbers in each GO term, x-axis in top indicated p-value of GO terms, y-axis indicated different GO terms.


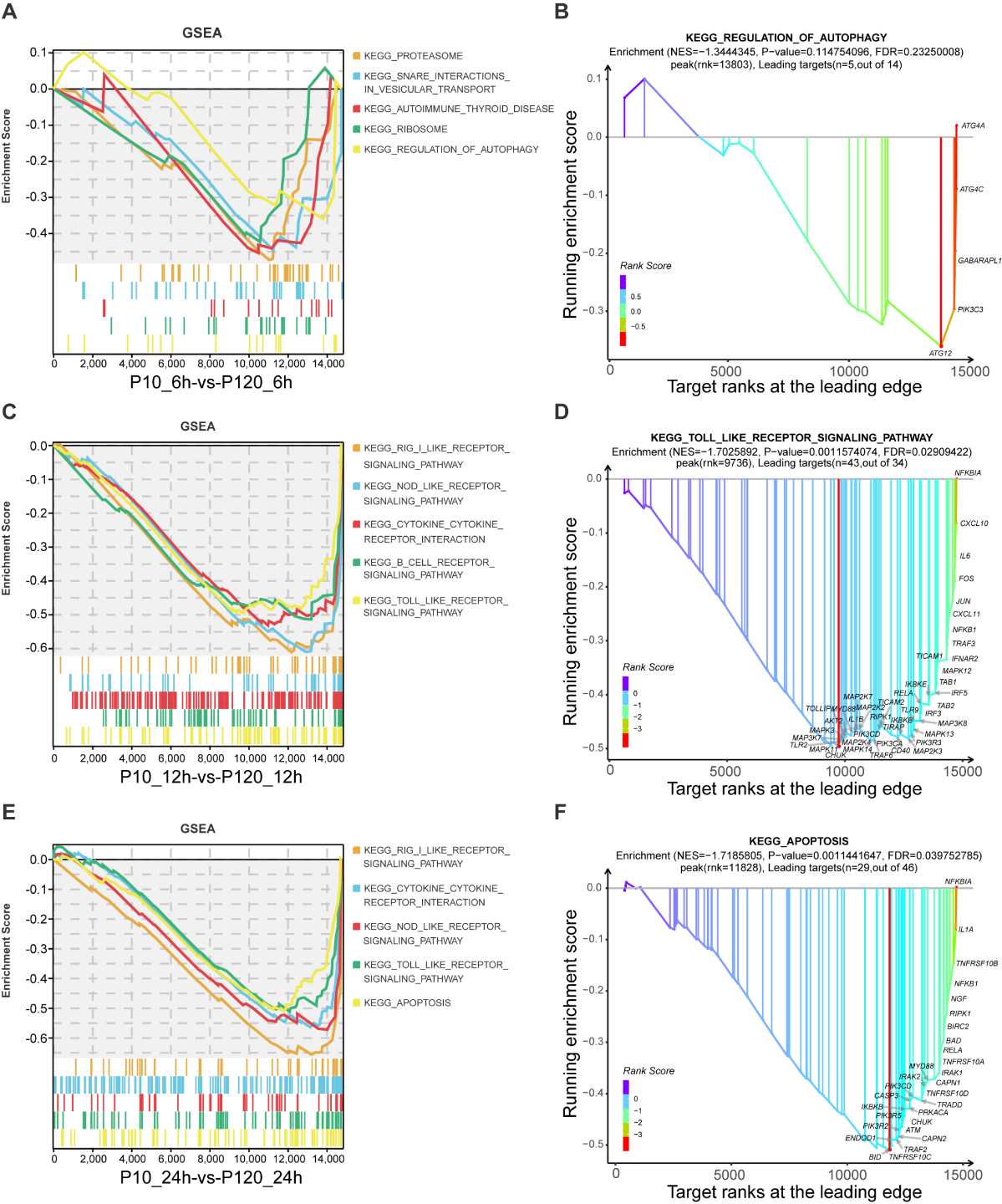


Figure 6

Top 5 enriched viral infection- or immune-related KEGG pathways in GSEA results. A, C, E, top 5 enriched viral infection- or immune-related KEGG pathways in GSEA results of CT-P120 infected cells versus CT-P10 infected cells at 6, 12, 24 hpi. B, D, F, top 1 enriched viral infection- or immune-related KEGG pathways with gene annotations in GSEA results of CT-P120 infected cells versus CT-P10 infected cells at 6, 12, 24 hpi.


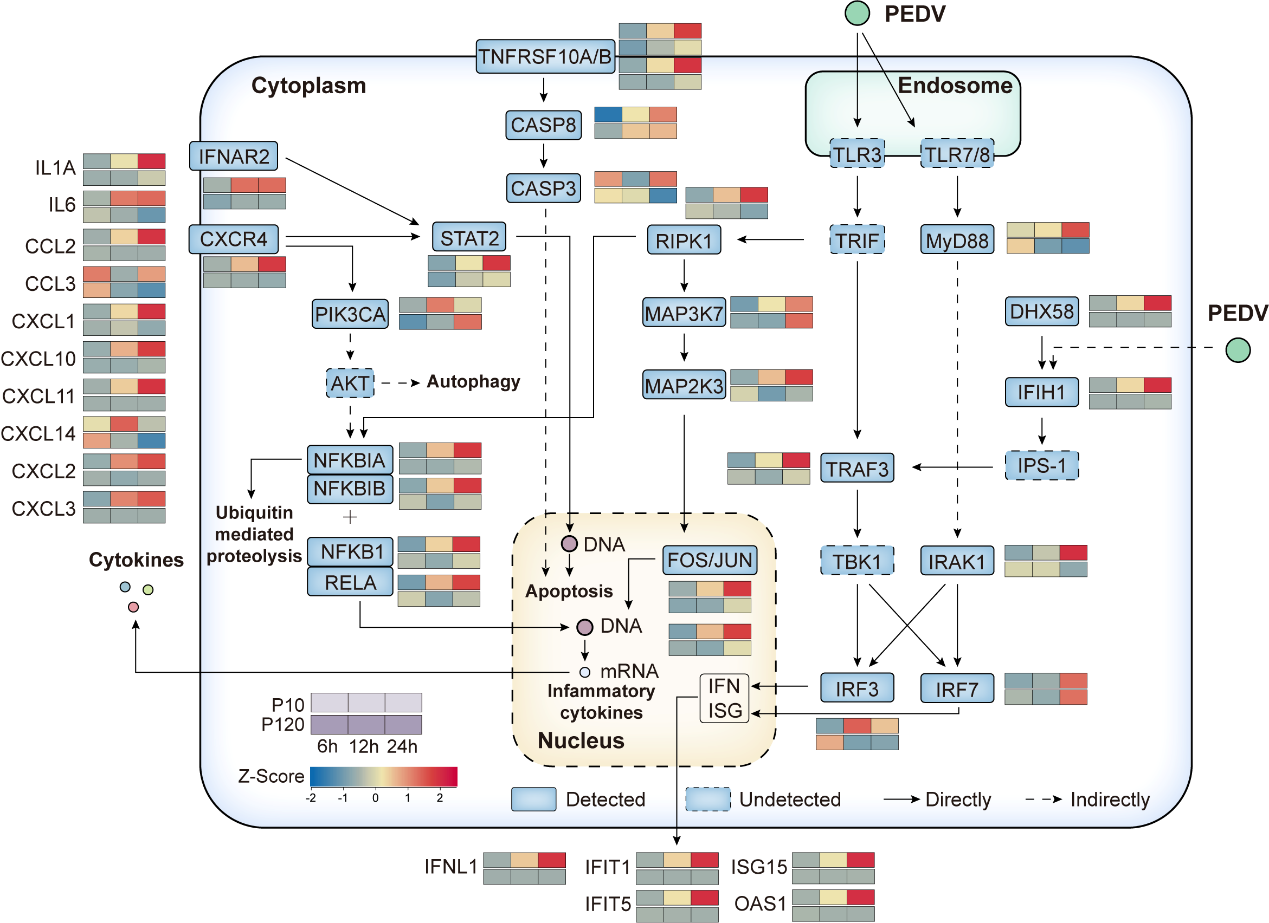


Figure 7

Map of genes in immune-related pathways with heatmaps involved in PEDV infection. Rounded rectangles with solid line indicate detected genes in transcriptomic data, rounded rectangles with dotted line indicated undetected genes in transcriptomic data. Heatmaps next to the rounded rectangles indicate the gene expression levels in a z-score form, the upper lanes indicate the CT-P10 infection group, the lower lanes indicate the CT-P120 infection group. Solid lines with arrows indicate the direct interaction, dotted lines with arrows indicate the indirect interaction.


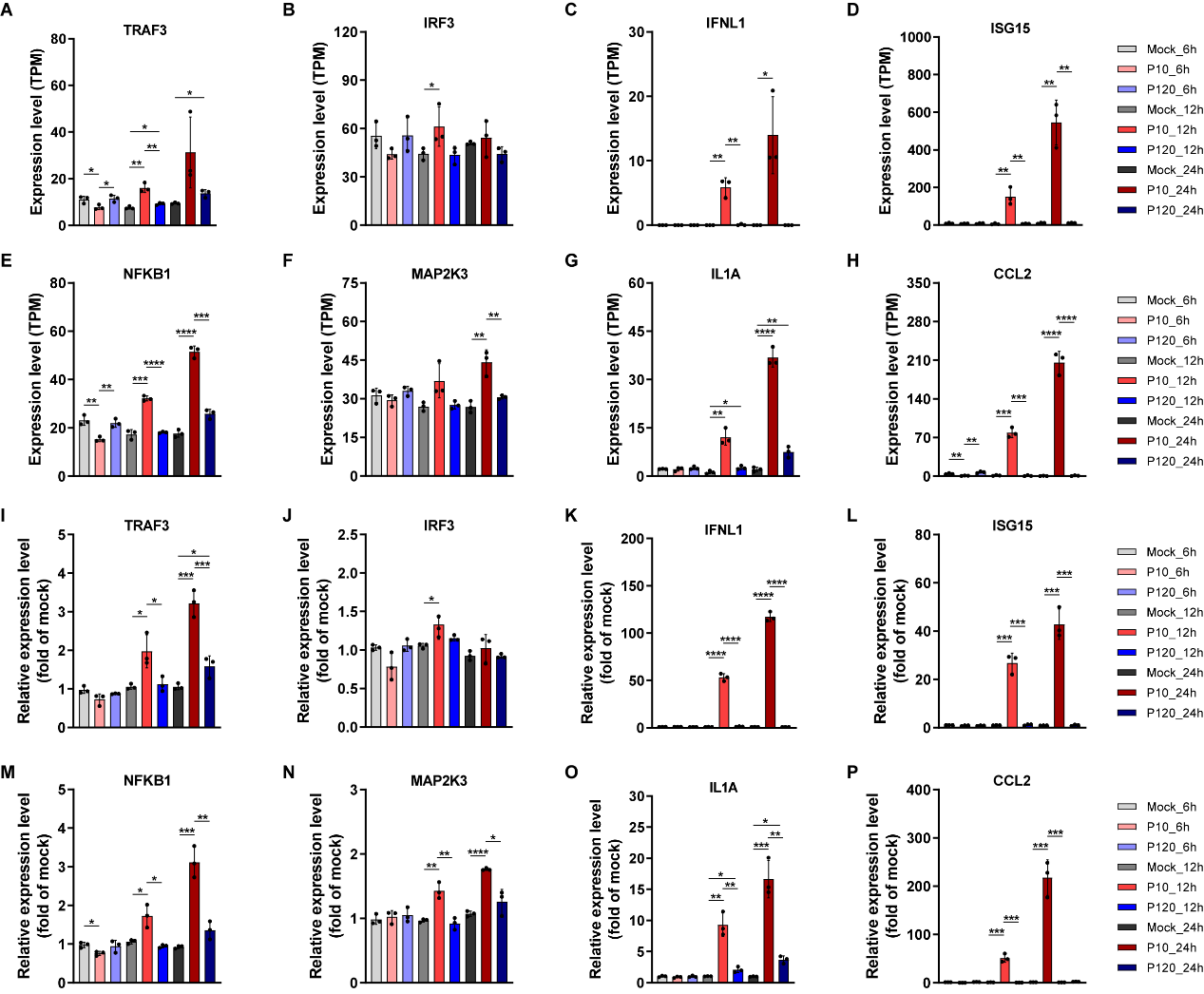


Figure 8

Validation of RNA-sequencing data by quantitative real-time PCR. Cultured Vero E6 cells were mock-infected or infected with PEDV strain CT-P10 or CT-P120 at MOI of 0.1. At 6, 12, and 24 hpi, samples were harvested and relative mRNA expression levels of indicated genes were measured by quantitative real-time PCR. A-H. Expression level of eight selected genes in transcriptomic data. I-P. Expression level of eight selected genes examined by RT-qPCR. Data are expressed as mean ​± ​SEM from three independent experiments. Data were analyzed using the Mann-Whitney test. *: P ​< ​0.05, ∗∗: P ​< ​0.01, ***: P ​< ​0.001, ****: P ​< ​0.0001. SEM, standard error of mean.


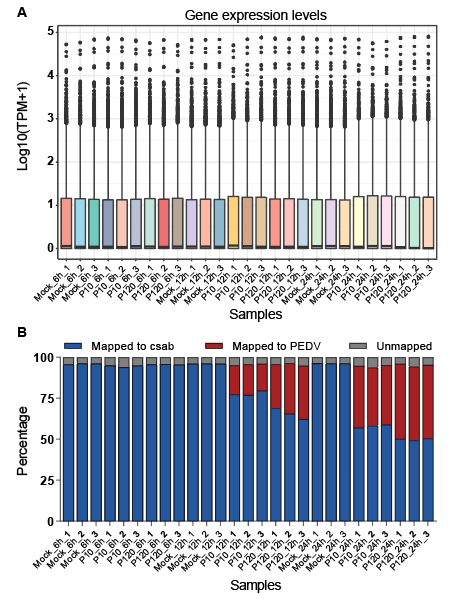


Supplementary figure S1

Basic information of sequencing reads. A. Box plot of gene expression levels. The x-axis is the name of the sample, and the y-axis is the value of the expression TPM after log 10 logarithmic processing. Each color in the figure represents a sample. B. Histogram of reads alignment distribution.


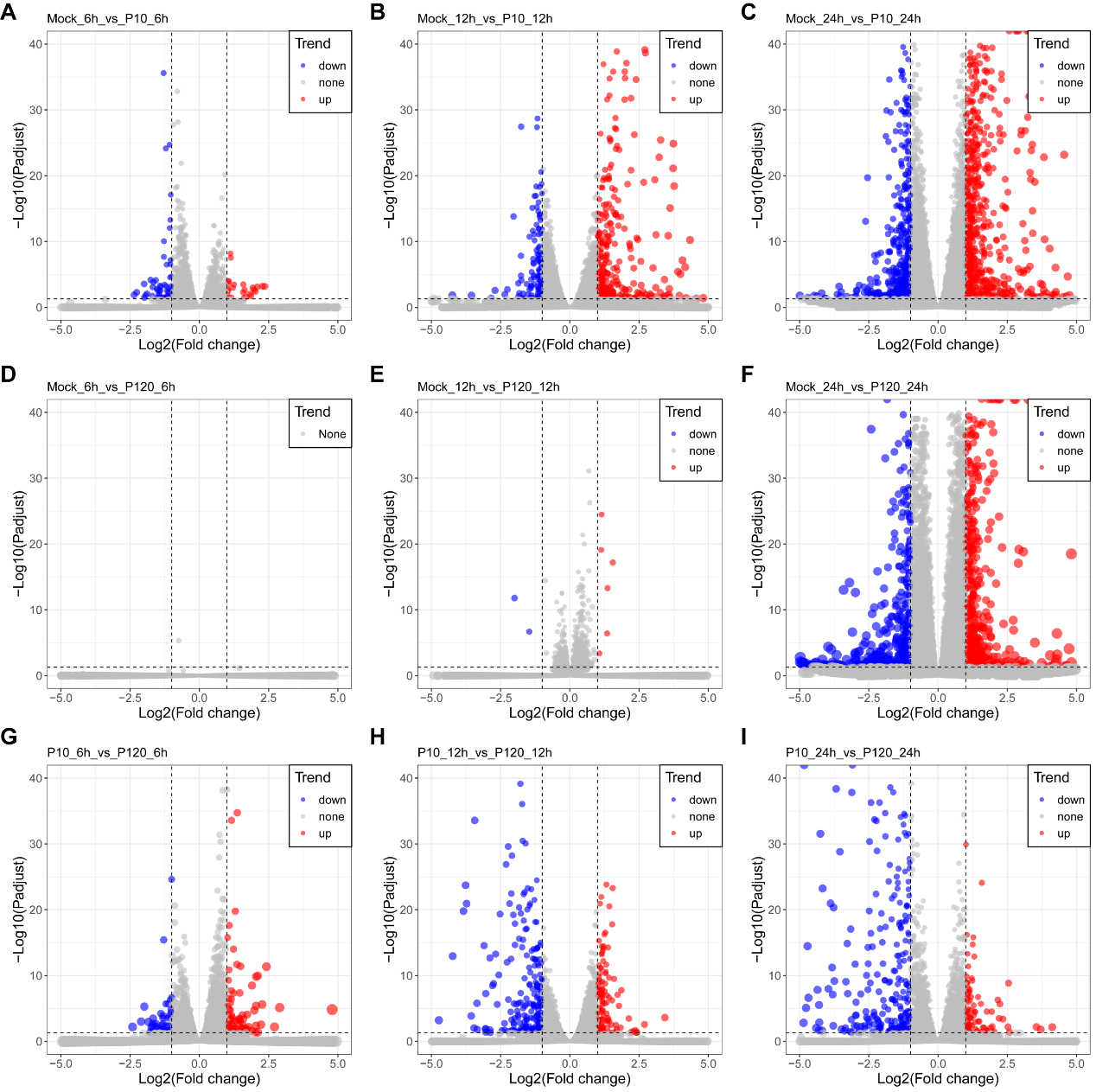


Supplementary figure S2

Volcano plots of DEGs. A-C. Volcano plots of DEGs between CT-P10 infected groups and mock-infected groups at 6, 12 and 24 hpi. D-F. Volcano plots of DEGs between CT-P120 infected groups and mock-infected groups at 6, 12 and 24 hpi. G-I. Volcano plots of DEGs between CT-P120 infected groups and CT-P10 infected groups at 6, 12 and 24 hpi.


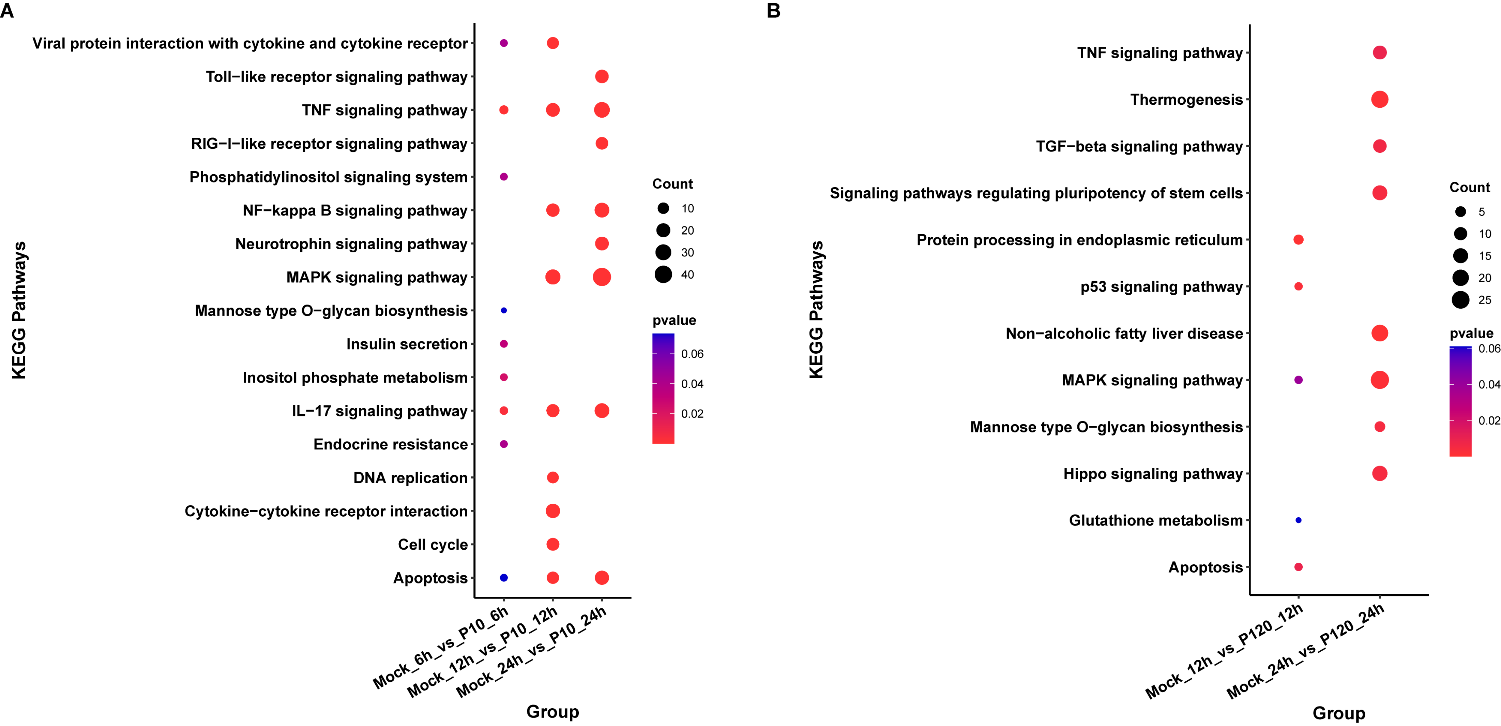


Supplementary figure S3

Bubble plots of KEGG pathways enriched with DEGs. A. Bubble plot of KEGG pathways in CT-P10 infected groups compared with Mock-infected groups. B. Bubble plot of KEGG pathways in CT-P120 infected groups compared with Mock-infected groups. The size of each point represents the number of DEGs, the color of each point represents the p-value of enriched KEGG pathways.


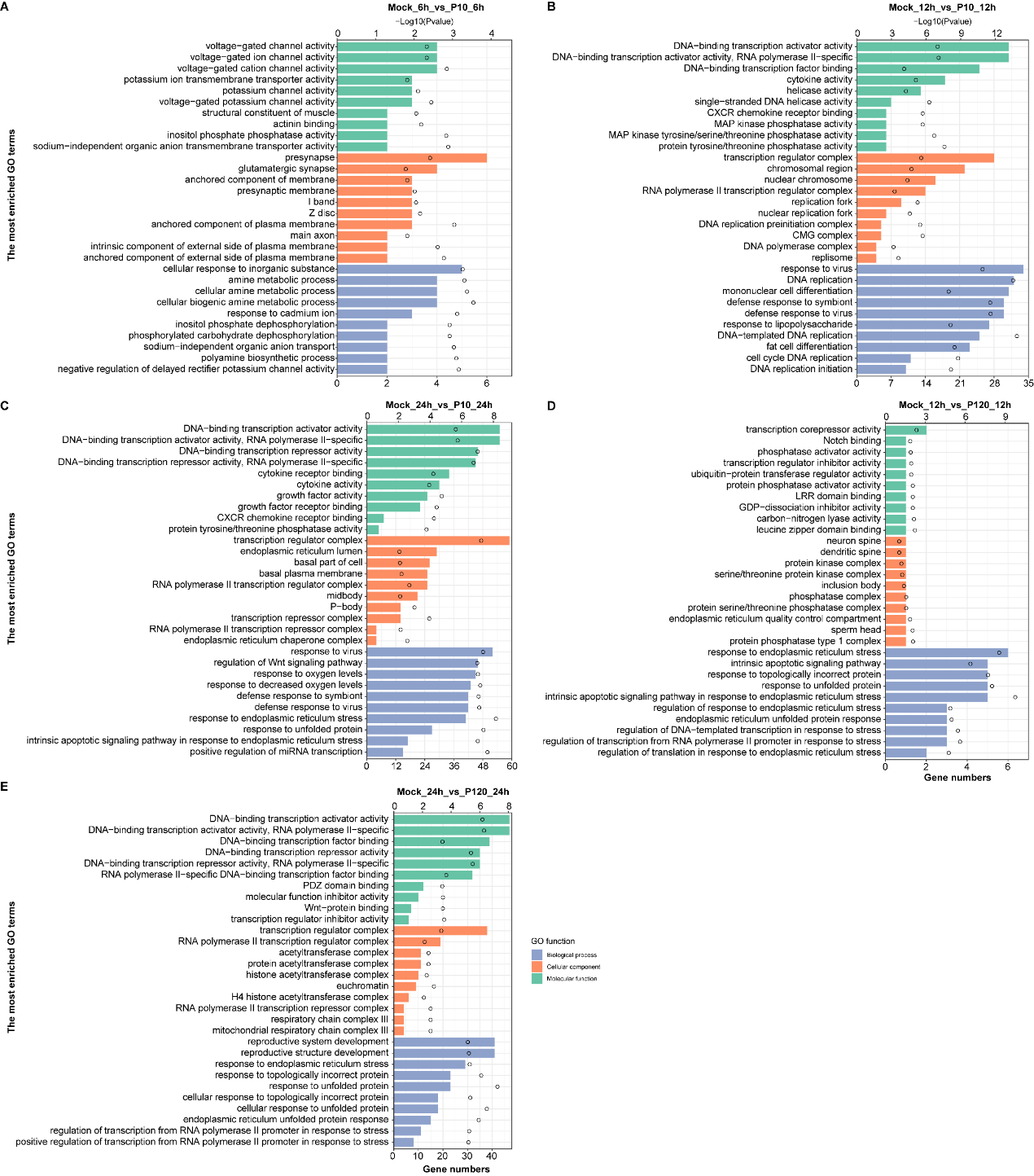


Supplementary figure S4

Bar plots of Gene Ontology (GO) terms enriched with DEGs. A-C. Bar plot of GO terms in CT-P10 infected groups compared with Mock-infected groups at 6, 12 or 24 hpi. D-E. Bar plot of GO terms in CT-P120 infected groups compared with mock-infected groups at 12 or 24 hpi. Bars in green indicated biological process terms, bars in orange indicated cellular component terms, bars in blue indicated molecular function terms. X-axis in bottom indicated gene numbers in each GO terms, x-axis in top indicated p-value of GO terms, y-axis indicated different GO terms.


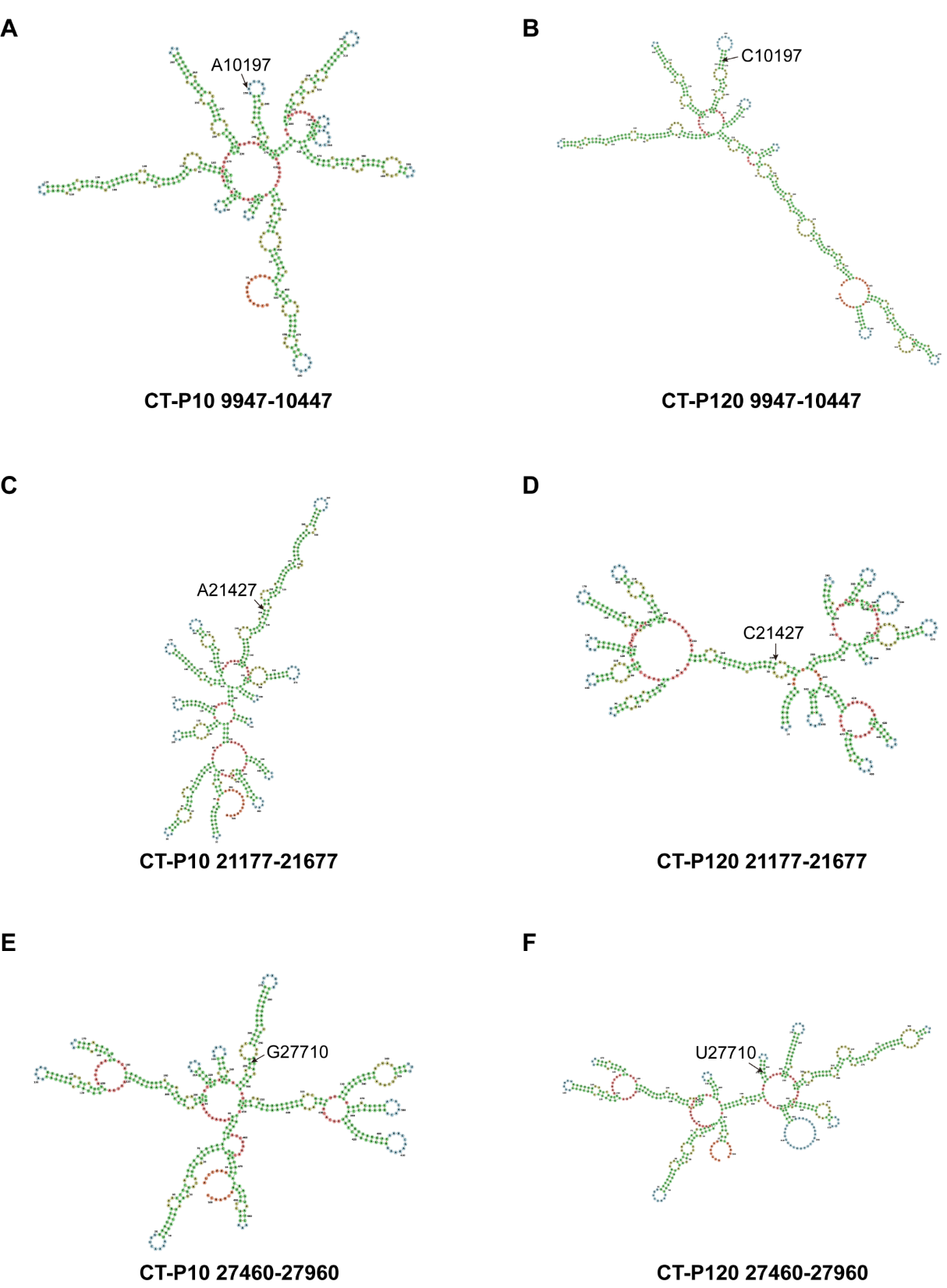


Supplementary figure S5

RNA secondary structure flanking the CT-P120 genome three mutation region (250 bp upstream and 250 bp downstream of mutation site). A, C and E. RNA secondary structure of CT-P10 genome flanking the mutation region at 10197, 21427 and 27710. B, D and F. RNA secondary structure of CT-P120 genome flanking the mutation region at 10197, 21427 and 27710.


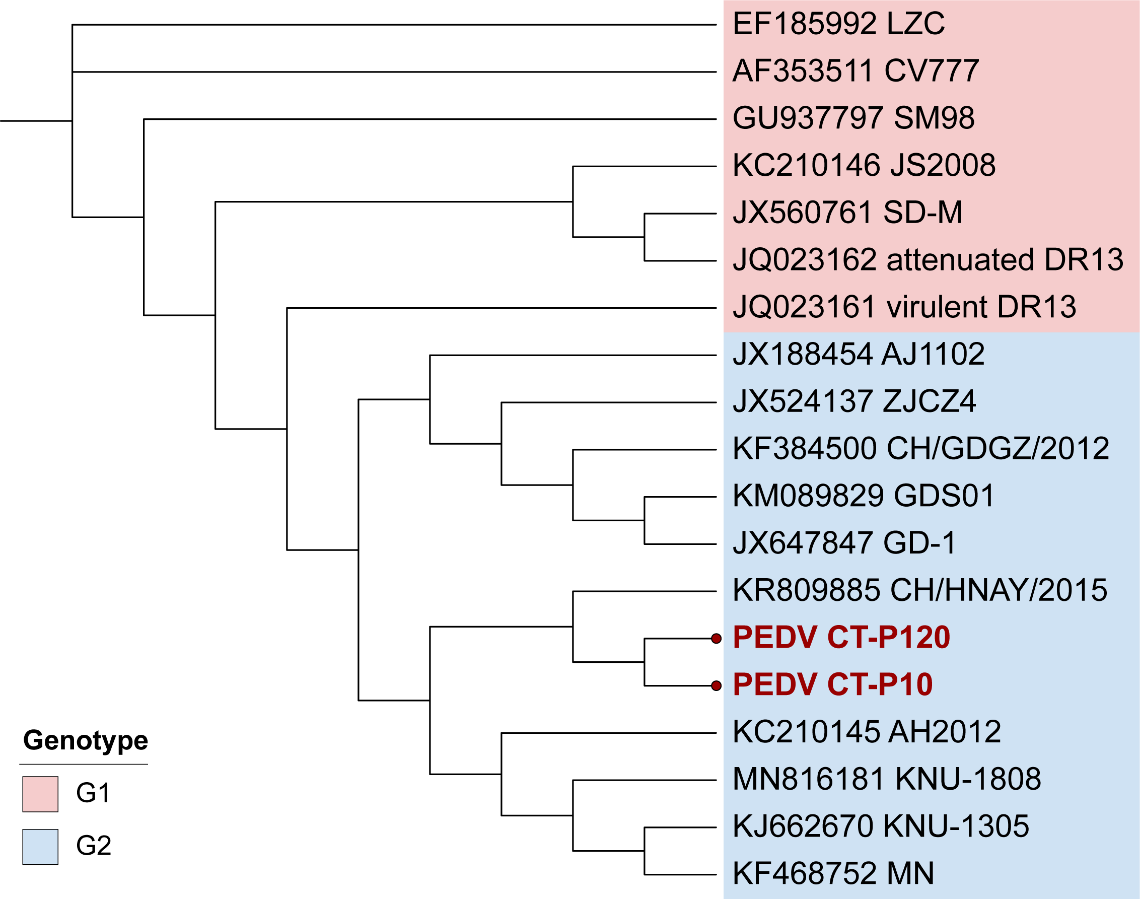


Supplementary figure S6

Phylogenetic analysis of CT-P10 and CT-P120 strains based on whole genome.
